# Minoxidil is a potential neuroprotective drug for paclitaxel-induced peripheral neuropathy

**DOI:** 10.1038/srep45366

**Published:** 2017-03-28

**Authors:** Yi-Fan Chen, Li-Hsien Chen, Yu-Min Yeh, Pei-Ying Wu, Yih-Fung Chen, Lian-Yun Chang, Jang-Yang Chang, Meng-Ru Shen

**Affiliations:** 1 Department of Pharmacology, College of Medicine, National Cheng Kung University, Taiwan; 2 Institute of Basic Medical Sciences, College of Medicine, National Cheng Kung University, Taiwan; 3 Department of Internal Medicine, College of Medicine, National Cheng Kung University, Taiwan; 4 Department of Obstetrics and Gynecology, College of Medicine, National Cheng Kung University, Taiwan; 5 Graduate Institute of Natural Products, College of Pharmacy, Kaohsiung Medical University, Kaohsiung, Taiwan; 6 National Institute of Cancer Research, National Health Research Institutes, Tainan, Taiwan; 7 Advanced Optoelectronic Technology Center, National Cheng Kung University, Taiwan

## Abstract

Chemotherapy-induced peripheral neuropathy (CIPN) is a common side effect of cancer treatment. No medication has been shown to be effective in the treatment of CIPN. This study aims to integrate the image-based high-content screening, mouse behavior models and mechanistic cell-based assays to discover potential neuroprotective drugs. Among screened compounds, minoxidil showed the most potent neuroprotective effect against paclitaxel, with regard to neurite outgrowth of dorsal root ganglia (DRG). Minoxidil protected mice from thermal insensitivity and alleviated mechanical allodynia in paclitaxel-treated mice. The ultrastructure and quantified G-ratio of myelin integrity of sciatic nerve tissues supported the observations in mouse behavioral tests. The mechanistic study on DRG neurons suggested that minoxidil suppressed neuroinflammation and remodeled the dysregulation of intracellular calcium homeostasis provoked by paclitaxel. Importantly, minoxidil showed a synergistic anti-tumor effect with paclitaxel both in tumor xenograft models of cervical and breast cancer. Interestingly, the quantitative assays on hair length and hair growth both exhibited that minoxidil significantly improved the hair quality after chemotherapy. Since minoxidil is a drug approved by the Food and Drug Administration (FDA), the safety and biocompatibility are well documented. The immediate next step is to launch an early-stage clinical trial intending to prevent CIPN by minoxidil.

Chemotherapy is a major category of cancer treatment that uses with a curative intent or reduces symptoms. Chemotherapy causes common side effects, including nausea, vomiting, bone marrow suppression and nerve system disorder^1^,^2^,^3^. Most of the side effects can be prevented except the chemotherapy-induced peripheral neuropathy (CIPN). CIPN, varying from 30 to 70% of patients receiving chemotherapy, leads to poor quality of life and discontinuation of useful anti-cancer treatment^4^. For example, paclitaxel is one of commonly used chemotherapeutic agents for the treatment of breast, lung and ovarian cancers^5^,^6^. It disturbs microtubules dynamics and undergoes multipolar mitosis; hence, the cell cycle arrested and eventually caused the cancer cell death^7^,^8^. However, in the clinical practice, a considerable proportion of cancer survivors experienced the side effects of paclitaxel treatment, including loss of sensory sensitivity and hyperalgesia^9^,^10^.

There are no biomarkers to predict the risk of cancer patients who will be suffered from CIPN. Furthermore, no medication is approved to be effective in the prevention or treatment of CIPN in large randomized, placebo-controlled clinical trials. The molecular mechanisms of CIPN remain unknown. Using animal models of CIPN, several new strategies to prevent or treat CIPN are under development. These new strategies involve several biological functions, including ion channel activation, glutamatergic neurotransmission, oxidative stress, cannabinoid system, inflammation, and mitochondrial functions.

The development of appropriate pre-clinical assay models and objective assessments of chemotherapy-associated neurotoxicity are the critical steps to predict patient risk and test potential therapies for toxic reduction or prevention. We have previously developed an image-based high-content platform which could be used to screen for potential neuroprotective drugs against paclitaxel neurotoxicity in cortical neurons^11^. This high-content screening system comprised of automated image acquisition and multiparameter analysis of neuronal functions.

The dorsal root ganglia (DRG) are the main target of platinum drug–induced CIPN. In this study, by the model of DRG neurons, we integrated the high-content image platform, mouse behavior models and mechanistic cell-based assays to discover potential neuroprotective drugs from the drug library of ion channels. The results showed that minoxidil, a drug approved by the Food and Drug Administration (FDA) for hypertension and alopecia, alleviated paclitaxel-induced neuropathy partly from restoring [Ca^2+^]_i_ dysregulation and suppressing neuroinflammation. Moreover, minoxidil showed a synergistic anti-tumor effect with paclitaxel and improved the hair quality after paclitaxel treatment.

## Results

### Minoxidil is a potential neuroprotective agent by high-content screening

**Figure 1:**
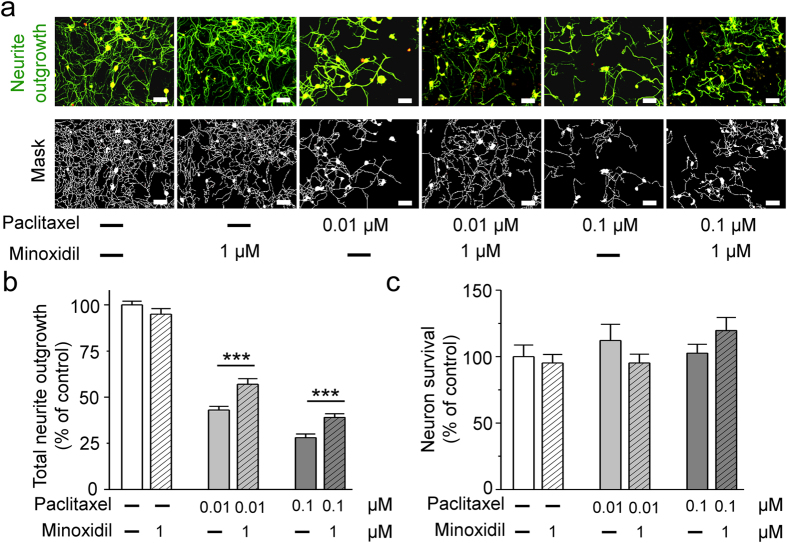
The neuroprotective effects of minoxidil on the neurite outgrowth of dorsal root ganglion (DRG) neurons. To test neuroprotective effects of minoxidil, primary culture of DRG neurons from 7-weeks-old C57/B6J mice were pre-treated with minoxidil for 24 hours and then exposed to 0.1 or 0.01 μM paclitaxel for another 24 hours. Minoxidil exhibited a significant neuroprotective effect against paclitaxel with regard to neurite outgrowth in DRG neurons. (**a**) Representative images show that minoxidil is a potential neuroprotective drug *in vitro* DRG neuron model. Scale bar, 50 μm. Green, anti-β-III tubulin antibody. Red, anti-NeuN antibody. (**b**) Quantitative analyses of neurite outgrowth in DRG neurons. **(c)** Quantitative analyses of neural viability in DRG neurons. Each value represents the mean ± S.E.M. from at least 5 different experiments. ***P < 0.001, compared with various concentration of paclitaxel-treated group, by two-tailed student’s t test.

DRG neurons are the main source of neuron damage in CIPN. Therefore, with the model of neurite outgrowth on DRG neurons, we first searched for potential neuroprotective agents by image-based high-content screening^11^. Paclitaxel was used as a neurotoxic drug to screen for potential neuroprotective agents from the compound libraries of ion channel ligands, REDOX and GABAergic ligands. We studied the toxic effect of paclitaxel on DRG neurons by analyzing two parameters including neurite outgrowth and neuron survival (Fig. 1). The main toxic effect of paclitaxel on DRG is to damage neurite outgrowth. The neuroprotective effect of minoxidil is to inhibit paclitaxel-induced damage. Minoxidil itself could not promote the neurite growth or neuron survival (Fig. 1b,c). As the Fig. 1b shown, 0.01 and 0.1 μM paclitaxel inhibited a 50% and 75% of DRG neurite outgrowth, respectively. Among 200-screened compounds, 1 μM minoxidil showed the most potent neuroprotective effect against paclitaxel, with regard to DRG neurite outgrowth (Fig. 1b).

### Minoxidil alleviates paclitaxel-induced neuropathy

Subsequently, mouse behavioral models of paclitaxel-induced neuropathy were used to study the potential neuroprotective effects of minoxidil. Previous studies have shown that minoxidil with 25 to 50 mg∙kg^−1^ was the maximal tolerable dose in the mouse model to study the antinociceptive effect on pain^12^,^13^. The drug concentrations used in this study mimic the oral administration of minoxidil for hypertension^14^. Hence, we did further experiments to monitor blood pressure, heart rate and locomotion during the procedure of treatment. As shown in Fig. S1, the hemodynamic stability was not affected by paclitaxel alone. Compared to the basal levels, low dose of minoxidil (25 mg∙kg^−1^) decreased 20–25% and 10–15% of systolic and mean blood pressure, respectively, at the initial 4-hour administration. One hour after drug injection, high dose of minoxidil (50 mg∙kg^−1^) induced tachycardia and significantly decreased blood pressure by 30–35%, which gradually returned to the basal level within 24 hours. In spite of the side effect on hemodynamic stability, minoxidil did not impair the locomotor activity within 24-hour treatment.

**Figure 2:**
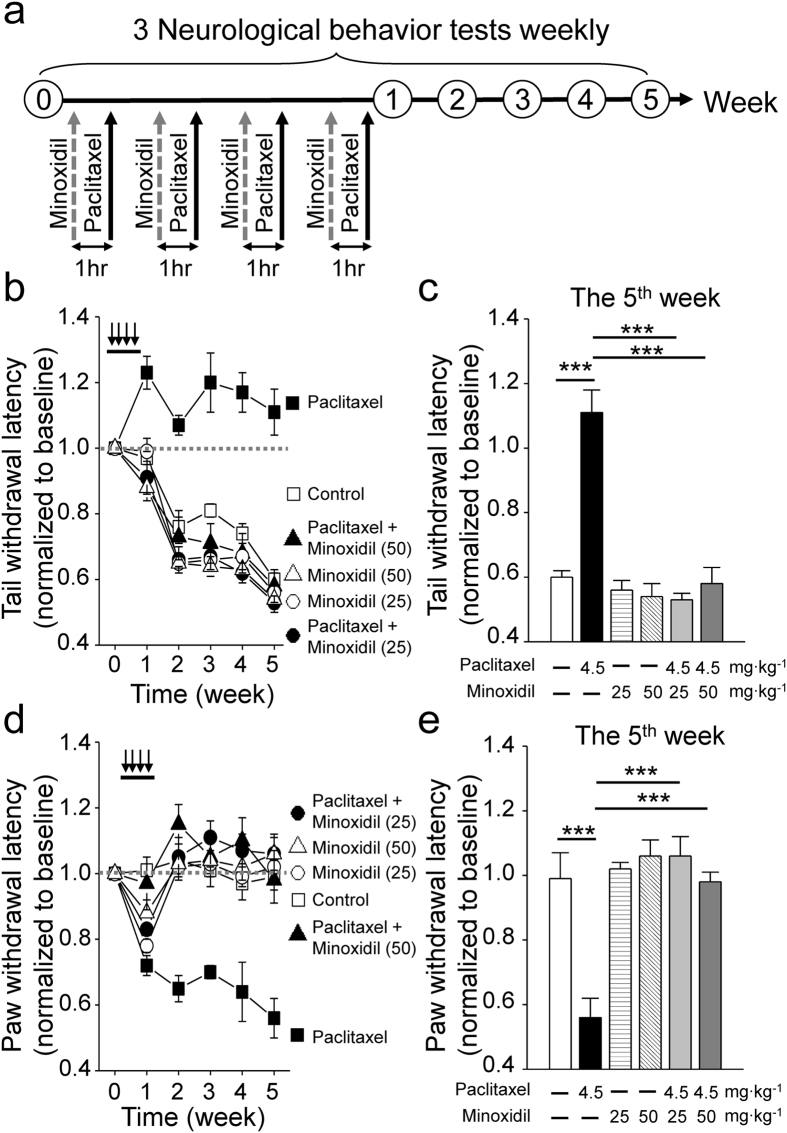
The neuroprotective effects of minoxidil in the mouse model. (**a**) Protocol showing drug administration and behavioral tests in the mouse model. The basal levels of each behavioral assay were obtained prior to the treatment. In the first week, 4.5 mg∙kg^−1^ paclitaxel was injected intraperitoneally every other day and vehicle or minoxidil (25 or 50 mg∙kg^−1^) was administrated by intraperitoneal injection one hour prior to paclitaxel treatment. After four courses of treatment, behavioral tests were done weekly. (**b**) Tail immersion test to assess thermal sensation. Y axis, normalized latency from tail immersion to tail withdrawal. Black arrow, drug infusion. (**c**) Quantitative analyses of tail immersion at the 5^th^ week. Each value represents mean ± SEM from at least 7 mice in each group. ***P < 0.001 versus paclitaxel treatment, by two-way ANOVA. (**d**) Von Frey filament test to detect allodynia. Y axis, normalized pressure from touch to paw withdrawal. Black arrow, drug infusion. (**e**) Quantitative analyses of von Frey filament test at the 5^th^ week. Each value represents mean ± SEM from at least 7 mice in each group. ***P < 0.001 versus paclitaxel treatment, by two-way ANOVA.

Here, 7 weeks-old C57BL/6J female mice were treated with 25 or 50 mg∙kg^−1^ minoxidil 1 hour prior to each injection of 4.5 mg∙kg^−1^ paclitaxel on 4 alternative days. Thermal threshold and mechanical allodynia for the assessment of small fiber dysfunction were weekly measured by tail immersion study and von Frey filament test, respectively (Fig. 2a). Increased thermal threshold (Fig. 2b,c) and decreased mechanical threshold (Fig. 2d,e) were observed in paclitaxel-treated mice, consistent with the clinical neurological findings of cancer patients treated with paclitaxel^15^. Pretreatment with minoxidil for total 4 dosages over 7 days protected mice from thermal insensitivity induced by paclitaxel (Fig. 2b,c). In addition, minoxidil significantly enabled mice to recover from paclitaxel-induced mechanical allodynia (Fig. 2d,e). Grip test was used to study the motor function (Fig. S2a and b). Consistent with previous studies^11^,^16^, mice with paclitaxel treatment exhibited normal grip strength, compared to control groups (Fig. S2a and b). Lower dose of minoxidil (5 mg∙kg^−1^) showed no protective effect on paclitaxel-induced neuropathy (Fig. S2c–f). These results suggest that minoxidil prevents the development of paclitaxel-induced sensory deficit.

We also designed the experiments to study whether minoxidil could reverse paclitaxel-induced neurotoxicity. Minoxidil was administered after neuropathic pain provoked by paclitaxel and mouse behavior tests were measured weekly (Fig. S3). Two weeks after paclitaxel injection, the mice exhibited an increase of thermal threshold (Fig. S3b) and a significant decrease of mechanical threshold (Fig. S3d). Treated with 25 or 50 mg∙kg^−1^ minoxidil enabled the mice to regain their thermal sensitivity (Fig. S3b and c). In addition, 25 mg∙kg^−1^ minoxidil partially alleviated mechanical allodynia but showed no effect on 50 mg∙kg^−1^ minoxidil treatment (Fig. S3d and e).

### Minoxidil decreases paclitaxel-induced peripheral nerve damages

**Figure 3:**
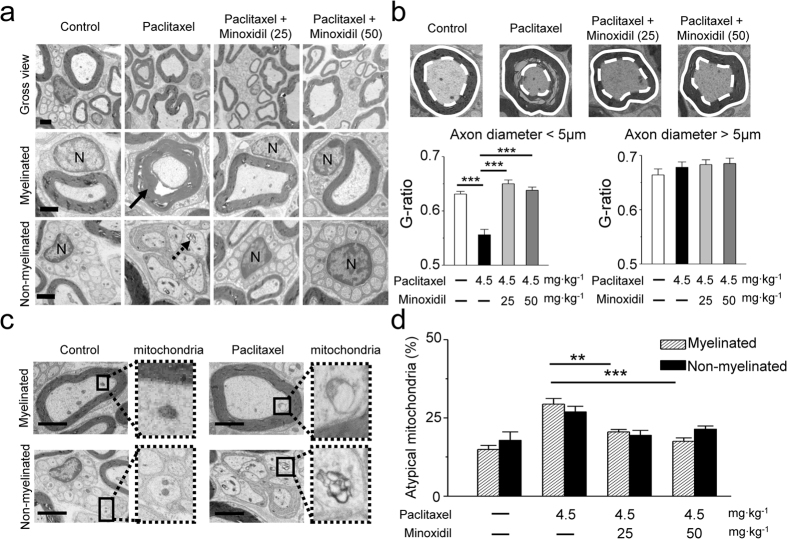
Ultrastructure of mouse sciatic nerve. (**a**) Transmission electron microscopy (TEM) images. Paclitaxel-induced sciatic nerve damage includes dying axons (black arrow), and swollen intra-axonal mitochondria (black dashed arrow). N, nucleus of Schwann cell. Scale bar, 1 μm. (**b**) G-ratio measurement in the sciatic nerves: the ratio of axon diameter (white dashed line) to myelin (white solid line). The G-ratio of small fibers (axon diameter <5 μm) and large fibers (axon diameter >5 μm) in mouse sciatic nerves from the four groups are presented. ***P < 0.001 versus paclitaxel treatment, by two-way ANOVA. (**c**) Representative TEM images show that paclitaxel caused swollen and vacuolated mitochondria (atypical mitochondria). Scale bar, 1 μm. (**d**) Quantitative analyses of atypical mitochondria indicate that minoxidil can prevent the mitochondrial damage caused by paclitaxel. Each value represents mean ± SEM of at least 100 mitochondria. **P < 0.01; ***P < 0.001 versus paclitaxel treatment, by two-way ANOVA.

Our mouse neurological studies clearly indicate that minoxidil has the protective effect against paclitaxel-induced neuropathy (Fig. 2). Accordingly, we examined the ultrastructure of sciatic nerves at the 5^th^ week after paclitaxel treatment (protocol as Fig. 2a). As shown in Fig. 3a, paclitaxel obviously induced axonal degeneration and demyelination (black arrow) in mouse sciatic nerves, in which nerve tissues displayed many swollen and vacuolated mitochondria in the axon (black dashed arrow). In contrast, pretreatment of minoxidil showed less severe axonal degeneration and myelin damage in sciatic nerve (Fig. 3a,b). Quantitative analysis of myelinated fiber densities revealed a significant decrease in paclitaxel-treated sciatic nerve axons compared to controls (P < 0.01). In contrast, pretreatment of 25 or 50 mg∙kg^−1^ minoxidil partially rescued paclitaxel-induced axon loss (Fig. S4). G-ratio, the ratio of inner axon circumference to outer myelin circumference, was utilized to measure the integrity of myelination. The representative micrographs (Fig. 3b) showed the axon (white dashed line) and myelin circumference (white circle) in myelinated fibers. We used axon sizes to differentiate small fibers (axon diameter <5 μm) from large fibers (axon diameter >5 μm). Paclitaxel induced a significant decrease in G-ratio of small fibers, and pretreatment of minoxidil remarkably attenuated this neurotoxic effect (P < 0.001, Fig. 3b). However, either paclitaxel or minoxidil did not affect the myelin integrity of large fibers (Fig. 3b). As depicted in Fig. 3c, we also found that paclitaxel caused enlarged and vacuolated mitochondria (atypical mitochondria) in sciatic nerve tissues. The number of atypical mitochondria was significantly decreased by the pretreatment of minoxidil (Fig. 3d). Taken together, the ultrastructure of sciatic nerve indicates the protective effect of minoxidil likely acts on both myelinated glial cells and neuron fibers of peripheral nerves, as evidenced by myelinated fiber densities (Fig. S4), the integrity of myelination by G ratio (Fig. 3b), and mitochondrial morphology in neuron fibers (Fig. 3c,d).

### Minoxidil augments anti-tumor effect of paclitaxel

**Figure 4:**
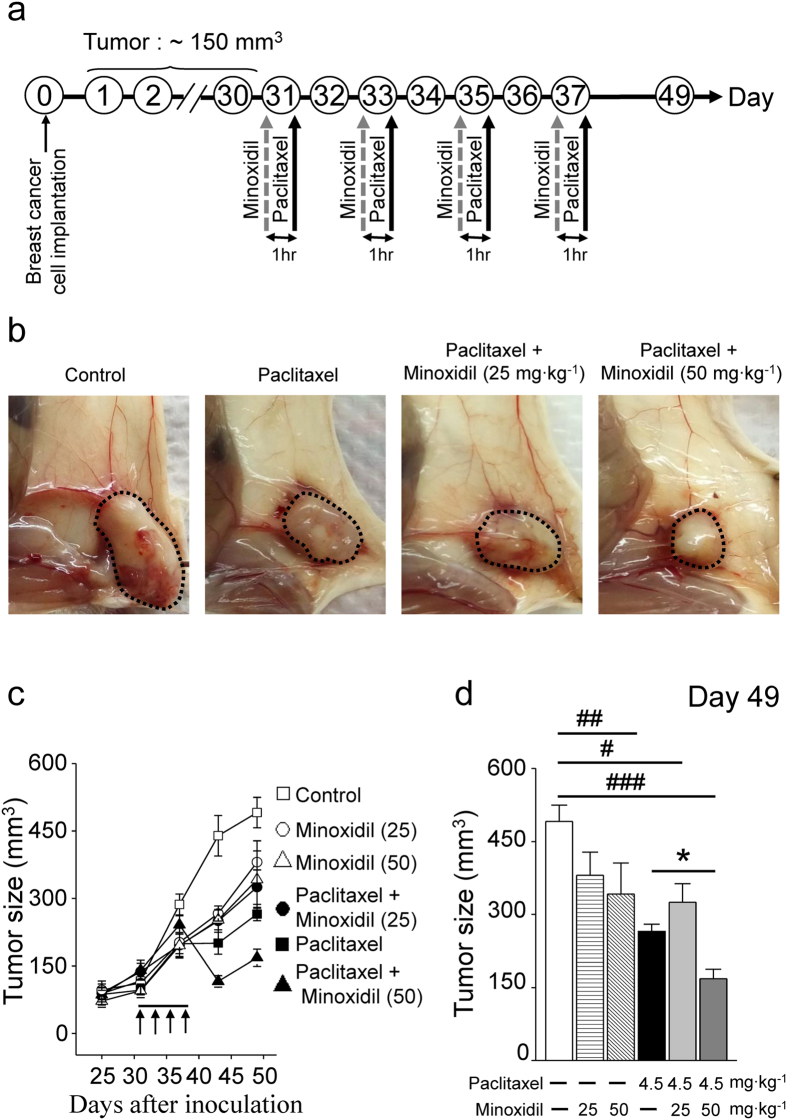
Minoxidil augments anti-cancer effect of paclitaxel. (**a**) Protocol showing the experimental design of tumor xenograft models of breast cancer. (**b**) Representative images showing minoxidil inhibits tumor growth and angiogenesis. Black dashed circle, breast adenocarcinoma. (**c**) Female NOD/SCID mice bearing tumor xenograft of breast cancer MDA-MB-231 cells were intraperitoneally injected every other day (arrows) with control saline, paclitaxel (4.5 mg∙kg^−1^) and minoxidil (25 or 50 mg∙kg^−1^) prior to paclitaxel treatment from the 31^th^ day post-inoculation (n = 5, per group). (**d**) Quantitative analyses of tumor volume at the day 49^th^ post-inoculation. Columns, mean ± SEM (n = 5, per group); ^#^P < 0.05; ^##^P < 0.01; ^###^P < 0.001 versus saline treatment; *P < 0.05 versus paclitaxel treatment, by two-way ANOVA.

We did the experiments to study the effect of minoxidil on tumor cell growth. As shown in Fig. S5, both minoxidil and paclitaxel inhibited cervical cancer SiHa cell proliferation by impairing cell viability. More importantly, they showed additive effect on inhibiting cancer cell proliferation. Furthermore, to test whether minoxidil alters tumor growth *in vivo*, we inoculated NOD/SCID mice subcutaneously with breast cancer MDA-MB-231 cells or cervical cancer SiHa cells. Mice were pretreated with minoxidil (25 or 50 mg∙kg^−1^) 1 hour prior to each injection of paclitaxel (4.5 mg∙kg^−1^) on four alternative days, when tumor volumes had reached a size of approximately 150 or 200 mm^3^ (Figs 4a and S6a). As depicted in Fig. 4b, rapid tumor growth was noted in the control groups. Paclitaxel alone or combined with minoxidil significantly inhibited tumor growth (Figs 4c,d and S6b,c). Minoxidil alone, 25 or 50 mg∙kg^−1^, also significantly inhibited the growth of cervical cancer (Fig. S6b and c). More importantly, high dosage of minoxidil (50 mg∙kg^−1^) showed a synergistic anti-tumor effect with paclitaxel.

### Minoxidil improves the hair quality after paclitaxel treatment

**Figure 5:**
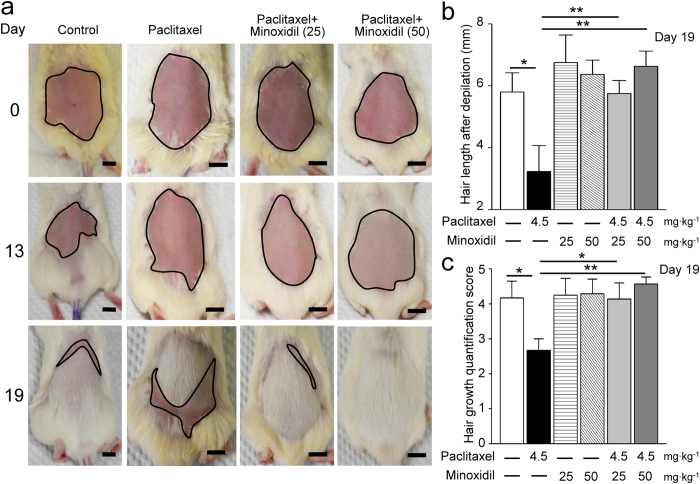
Minoxidil improves the hair quality after paclitaxel treatment. (**a**) Representative images showing hair re-growth in female NOD/SCID mice treated with vehicle, paclitaxel or minoxidil (25 or 50 mg∙kg^−1^) plus paclitaxel treatment for 0 (upper panel), 13 (middle panel) and 19 days (lower panel). Black circle, shaved skin. Scale bar, 1 cm. (**b**) Re-growth of hair was quantified by measuring the length of plucked hairs at Day 19 after epilation. Each value represents mean ± SEM of at least 10 hairs. *P < 0.05; **P < 0.01 versus paclitaxel treatment, by two-way ANOVA. (**c**) Visual scoring of the hair growth by the scoring guideline shown in Fig. S7b. Each value represents mean ± SEM of at least 6 mice. *P < 0.05; **P < 0.01 versus paclitaxel treatment, by two-way ANOVA.

Hair loss, also called alopecia, is a common and distressing side effect of chemotherapy, which may occur throughout the body, including the head, face, arms, legs, underarms, and pubic area^17^,^18^. Considering minoxidil is a well-known FDA approved drug for alopecia, we studied whether minoxidil could improve hair growth after paclitaxel treatment in mouse models. As the protocol shown in Fig. S7a, after epilation by hair removal cream, NOD/SCID female mice were pretreated with minoxidil (25 or 50 mg∙kg^−1^) 1 hour prior to each injection of paclitaxel (4.5 mg∙kg^−1^) over 7 days. The hair quality by measuring hair length and hair-covered area was scored on the 13^th^ and 19^th^ day after epilation, respectively (Fig. S7b). In the mice pretreated with minoxidil (25 or 50 mg∙kg^−1^), most of the shaved skin was covered by white and short hair on the 13^th^ days of epilation, which was strikingly contrast to the hairless skin of paclitaxel-treated mice (Fig. 5a). Furthermore, in the both groups of minoxidil pretreatment, the shaved areas were almost covered by hair on the 19^th^ day. More importantly, the quantitative assays on hair length and hair growth both show that minoxidil significantly improved the hair quality after chemotherapy (Fig. 5b,c).

### Minoxidil prevents [Ca^2+^]_i_ dysregulation induced by paclitaxel

**Figure 6:**
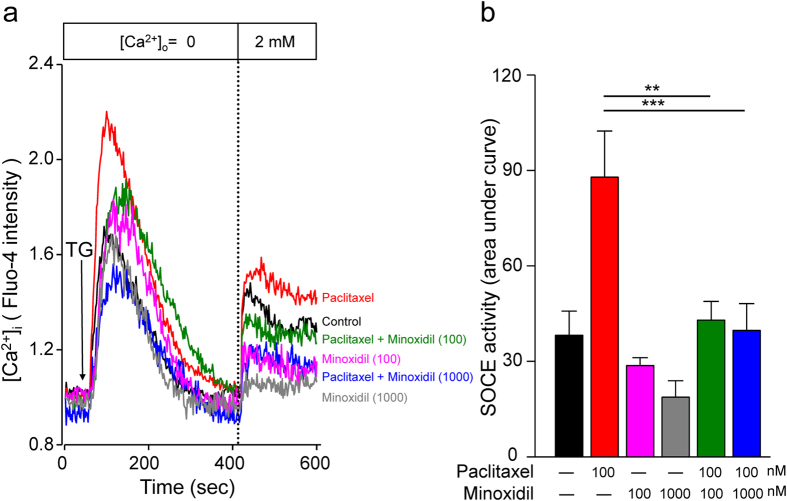
Minoxidil protects neurons by remodeling [Ca^2+^]_i_ homeostasis. (**a**) [Ca^2+^]_i_ measurement of SOCE amplitude in dorsal root ganglion cell line ND7/23. Mean traces of [Ca^2+^]_i_ measurement from at least 100 different ND7/23 cells in each experiment. The SOCE amplitude indicates the rise of [Ca^2+^]_i_ in replenishment of extracellular Ca^2+^ ([Ca^2+^]_o_) from 0 to 2 mM. Arrow, adding 2 μmol∙L^−1^ thapsigargin (TG). (**b**) Y axis, quantitative analyses of SOCE changes in Ca^2+^ level ([Ca^2+^]_i_). Each value represents mean ± SEM of at least 100 neurons. **P < 0.01; ***P < 0.001 versus paclitaxel treatment, by two-way ANOVA. SOCE: Store-operated Ca^2+^ entry.

According to the results of mouse behavioral tests and tumor xenograft models, minoxidil displays the neuroprotective effect against paclitaxel-induced neuropathy and has the synergistic anti-tumor effect with paclitaxel. We then studied the protective mechanism of minoxidil on neuropathy. Previous studies have shown that paclitaxel dysregulates store-operated calcium entry (SOCE) that partly contributes the neurological pain^11^,^19^. We used the DRG neuron cell line (ND7/23) to study the activation of SOCE in various conditions. In the [Ca^2+^]_i_ measurement of DRG neurons, the amount of Ca^2+^ influx following thapsigargin-induced endoplasmic reticulum (ER) store depletion, also called SOCE, was increased after paclitaxel treatment for 24 hours (Fig. 6). Importantly, minoxidil significantly reversed the paclitaxel-induced dysregulation of SOCE. This result can partly explain the protective effect of minoxidil on paclitaxel-induced peripheral neuropathy.

### Minoxidil suppresses paclitaxel-induced neuroinflammation

**Figure 7:**
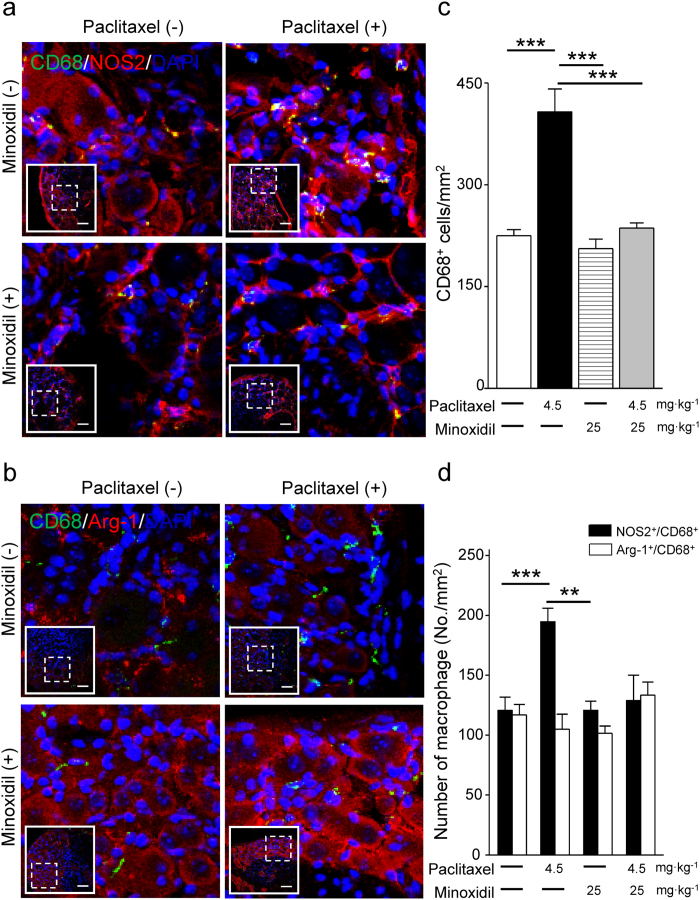
Paclitaxel induces M1 dominant macrophage recruitment in DRG neurons which is inhibited by minoxidil. (**a**) Representative images of immunofluorescent detection of CD68 (macrophage marker; green), NOS2 (M1-like macrophage marker; red), and (**b**) CD68 (macrophage marker; green), Arg-1 (M2-like macrophage marker; red) in DRG neurons at the 7^th^ day after first paclitaxel (4.5 mg∙kg^−1^) or minoxidil (25 mg∙kg^−1^) prior to paclitaxel treatment. Inserts show the lower magnification of DRG sections and the dash squares represent the enlarged view. Scale bar in (a) and (b), 50 μm. (**c**) The density of macrophage and (**d**) the number of NOS2^+^/CD68^+^ and Arg-1^+^/CD68^+^ macrophages were analyzed in DRG neurons at the 7^th^ day after paclitaxel treatment. Each value represents mean ± SEM from at least 5 different samples. **P < 0.01; ***P < 0.001 versus paclitaxel treatment, by two-way ANOVA.

**Figure 8:**
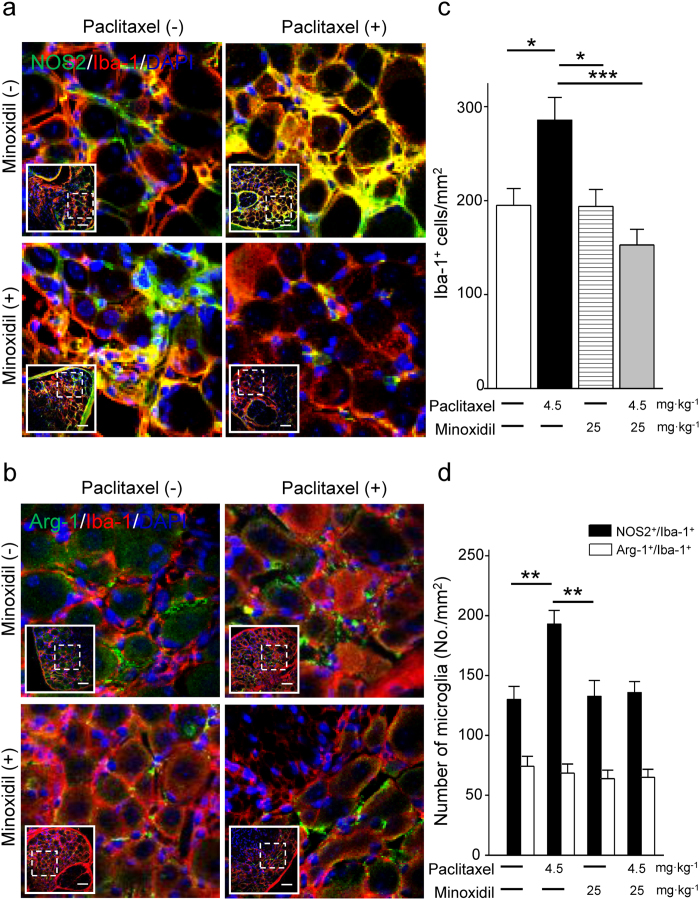
Paclitaxel induces M1 dominant microglia recruitment in DRG neurons which is inhibited by minoxidil. (**a**) Representative images of immunofluorescent detection of Iba-1 (microglia marker; red), NOS2 (M1-like macrophage marker; green), and (**b**) Iba-1 (microglia marker; red), Arg-1 (M2-like macrophage marker; green) in DRG neurons at the 7^th^ day after first paclitaxel (4.5 mg∙kg^−1^) or minoxidil (25 mg∙kg^−1^) prior to paclitaxel treatment. Inserts show the lower magnification of DRG sections and the dash squares represent the enlarged view. Scale bar in (**a**,**b**), 50 μm. (**c**) The density of microglia and (**d**) the number of NOS2^+^/Iba-1^+^ and Arg-1^+^/Iba-1^+^ microglia were analyzed in DRG neurons at the 7^th^ day after paclitaxel treatment. Each value represents mean ± SEM from at least 5 different samples. *P < 0.05; **P < 0.01; ***P < 0.001 versus paclitaxel treatment, by two-way ANOVA.

Paclitaxel induced macrophage recruitment and activation in DRG neurons, which is an evidence of neuroinflammation and contributes to peripheral neuropathic pain^20^,^21^. To study if minoxidil could prevent the paclitaxel-induced neuroinflammation, we did immunofluorescence analysis of mouse DRG neurons labeling with macrophage marker CD68. We first demonstrated that paclitaxel treatment activated macrophage recruitment to DRG neurons with a time-dependent manner, which reached a peak level on the 7^th^ day after paclitaxel treatment (Fig. S8c). Minoxidil alone did not affect the macrophage infiltration in DRG neurons on the 7^th^ day of injection (Fig. S9b). Importantly, 25 mg∙kg^−1^ treatment of minoxidil prior to each paclitaxel injection abolished paclitaxel-induced macrophage recruitment in DRG neurons (Fig. S9). We further identified which phenotype of macrophage recruitment was inhibited by minoxidil treatment. As shown in Figs 7 and 8, double staining of mouse DRG with macrophage and microglia markers reveals that the expression of NOS2^+^/CD68^+^ and NOS2^+^/Iba-1^+^ cells were significantly increased 7 days after paclitaxel treatments. However, macrophages expressing M2 phenotypic markers (Arginase-1) in CD68 or Iba-1 positive cells were unchanged relative to vehicle control group (Figs 7d and 8d). Paclitaxel induced macrophage recruitment in DRG neurons, in which activated M1 macrophage was the dominant group. This indicates that paclitaxel leads to an M1-like state that activates the pro-inflammatory cascades. Minoxidil itself did not affect the macrophage infiltration in DRG neurons (Figs S9b, 7c and 8c). Importantly, minoxidil treatment prior to paclitaxel injection abolished paclitaxel-induced macrophage recruitment in DRG neurons, especially inhibiting the proportion of NOS2^+^/CD68^+^ and NOS2^+^/Iba-1^+^ M1 macrophages (Figs 7d and 8d). These results imply that minoxidil plays a role in the suppression of paclitaxel-induced neuroinflammation.

## Discussion

With the growing number of cancer survivors, it is important to consider toxicities that results from treatment and do not resolve after treatment ends. Some symptoms continue to burden patients for years after the cancer has been cured, and CIPN is a conspicuous example. Unfortunately, there is no medication approved to be effective in the prevention or treatment of CIPN. We previously identified hit compounds of neuroprotection from drug library by high-content image-based screening. With the screening model of cortical or DRG neurons, minoxidil, one of hit compounds showed the significant neuroprotective effects against paclitaxel-induced neurotoxicity^11^. Here we further demonstrated that minoxidil protected paclitaxel-induced peripheral neuropathy by integrating the mouse behavior models with mechanistic cell-based assays. This conclusion is supported by the mouse behavioral tests showing that minoxidil protected mice from thermal insensitivity and alleviated mechanical allodynia in paclitaxel-treated mice. Moreover, the ultrastructure and quantified G-ratio of myelin integrity of sciatic nerve tissues isolated from minoxidil/paclitaxel-treated mice supported the observations in mouse behavioral tests. More importantly, higher dosage of minoxidil (50 mg∙kg^−1^) showed a synergistic anti-tumor effect with paclitaxel both in NOD/SCID mice model of breast cancer and cervical cancer.

The drug concentrations used in this study mimic the oral administration of minoxidil for hypertension. Following oral administration of 2.5 to 10 mg, the plasma concentration of minoxidil ranges from 0.12 to 3.5 μM in human subjects^14^. In the mouse model for anti-nociceptive effect, the 25 and 50 mg∙kg^−1^ *in vivo* dose is equivalent to the dosage of 2.03 and 4.05 mg in human subjects^12^, respectively. To mimic the pharmacokinetics and pharmacodynamics in human subjects, we chose minoxidil at the concentration of 0.1 and 1 μM to test the biological effects in cell models. The dosage of the 25 or 50 mg∙kg^−1^ was used in mouse models. In the mouse model, high dose of minoxidil using in neuroprotective purpose showed the side effect on blood pressure and heart rate. This suggests that the safety on hemodynamic stability in using minoxidil to prevent CIPN should be monitored in the future clinical trials.

A considerable proportion of the cancer patients suffer from hair loss after chemotherapy^22^. Minoxidil was first developed to act as antihypertension drug, but the people were found to have the side effect of hypertrichosis, an abnormal amount of hair growth over the body^23^. Previous studies reported some possible mechanisms of minoxidil to promote the hair growth: (i) minoxidil increase the hair follicle size, reduction on telogen follicles and increase the proportion of follicles in anagen^24^; (ii) minoxidil promotes hair growth via suppress androgen receptor-related functions^25^; (iii) minoxidil stimulates cell growth, delay cell senescence^20^,^26^ and the stimulation of VEGF and prostaglandin synthesis^27^. Although, the mechanism of minoxidil action on hair growth is still unclear, it became a common medication for alopecia treatment. Interestingly, our data suggest that minoxidil can promote the hair growth after paclitaxel treatment.

Minoxidil is a K^+^ channel opener, which enhances K^+^ permeability and sequentially opposes the entry of Ca^2+^ into cells^28^. Nociception processing starts from the activation of peripheral sensory neurons by elevating intracellular Ca^2+^ concertation^29^. Previously, the activation of K^+^ channels by minoxidil has been as drug targets for nociceptive pain^12^. Similarly, paclitaxel gradually increases the intracellular Ca^2+^ release in cultured DRG, which partly contributes to the pathogenesis of neuropathic pain^30^. Our results showing that pretreatment with minoxidil significantly reversed the paclitaxel-evoked intracellular Ca^2+^ concentration suggests that minoxidil may prevent CIPN by mediating [Ca^2+^]_i_ homeostasis in DRG neurons. In addition, neuropathic pain is associated with excessive inflammation in peripheral nerve system that leads to persistent pain^31^. Paclitaxel induced macrophage infiltration into DRG tissues and caused to develop peripheral neuropathic pain^32^. Consistent with previous studies, the macrophage recruitment in DRG was increased on the 7^th^ day after the first injection of paclitaxel. Treatment of minoxidil prior to each paclitaxel injection can suppress paclitaxel-induced neuroflammation, as evidenced by the decreased infiltration of M1 macrophages in DRG. Accordingly, there are two possible mechanisms to explain the protective effect of minoxidil on CIPN: one is to regulate [Ca^2+^]_i_ and the other is to suppress neuroflammation.

In conclusion, we demonstrate that minoxidil is a potential neuroprotective drug for paclitaxel-induced neuropathy. Since minoxidil is a FDA approved drug for hypertension and alopecia, the safety and biocompatibility are well documented. The immediate next step is to launch an early-stage clinical trial intending to prevent CIPN by administration of minoxidil.

## Methods

### Animals

The 6-weeks-old C57BL/6J and NOD/SCID female mice were maintained in the pathogen-free facility of the Animal Laboratory of National Cheng Kung University. These animals were housed in a temperature- (temperature: 25 ± 2 °C; humidity: around 60–80%) and light-control environment under a 12:12 h light-dark cycle (lights on at 6:00 AM) with free access to food and water according the approved guidelines by the Institutional Animal Care and Use Committee (IACUC) of National Cheng Kung University. Before carry out any experiments, all animals adapted to the housing environments for at least 7 days.

### Cell culture

For primary culture, DRGs were removed from 7-weeks-old C57BL/6 J mice, then digested with 0.1% collagenase (Sigma-Aldrich, USA) for 1 hour and followed by 0.25% trypsin for 25 minutes. The dissociated neuron cultures were plated at a density of 2.5 × 10^4^ cells per well in a 96 well optics plates (CLS3614; Sigma-Aldrich, USA) pre-coated with 1 mg∙mL^−1^ poly-D-lysine (A-003-M; Sigma-Aldrich, USA) for 30 mins. Primary DRG cells were obtained in DMEM-F12 (11320033; Gibco, USA) supplemented with 10% fetal bovine serum (FBS, 26140079; Gibco, USA). These cells were maintained at 37 °C in an environment containing 5% CO_2_. DRG cell line (ND7/23) was obtained from Sigma-Aldrich (92090903, USA) since 2015. The human breast adenocarcinoma (MDA-MB-231) and human cervical squamous cell carcinoma (SiHa) were authenticated by the short-tandem repeats analysis using the Promega StemElite ID System (GeneLabs Life Science Corp, Taiwan). MDA-MB-231 cells were maintained in RPMI-1640 (22400105; Gibco, USA) supplemented with 10% FBS. ND7/23 DRG cells and SiHa cells were grown in Dulbecco’s Modified Eagle Medium (DMEM, 12100046; Gibco, USA) supplemented with 2 mmol·L^−1^ L-glutamine and 10% FBS.

### High-content neuroprotective drug screening

Compound libraries of ion channel ligands were obtained from Enzo drug bank (BML-2805; ENZO Life Sciences, NY, USA). Primary mouse DRG neurons were incubated with compounds at final concentration of 1 μM for 24 hours, and then with 0.01 or 0.1 μM paclitaxel for another 24 hours. After paclitaxel treatment, primary DRG cells for neurite outgrowth analysis were washed by PBS and then fixed with 4% paraformaldehyde (PFA, P6148; Sigma-Aldrich) for 15 mins. These fixed DRG cells were washed with 0.05% Triton X-100 in PBS for 30 minutes and blocked with 3% bovine serum albumin (BSA; Sigma-Aldrich, USA) at room temperature for 1 hour. Subsequently, DRG cells were stained with goat anti-*β* III tubulin (SC-9935, 1:100; Santa Cruz, USA) and mouse anti-NeuN monoclonal antibody (1:100; Millipore, USA) overnight at 4 °C. The samples were then washed by PBS and reacted with secondary antibodies (Alexa-488 and Alexa-594 1:200; Invitrogen, CA) at room temperature for 1 hour. For image acquisition and analysis of DRG neurite outgrowth, images of stained cells were automatically acquired using 20X objective by ImageXpress^Micro^ wide field fluorescent microscope (Molecular Devices, USA) as previously described^11^.

### Mouse behavioral models of paclitaxel-induced peripheral neuropathy

Following housing adaptions, 7-weeks-old C57BL/6J female mice (weight range: 18–20 g) were used for studying paclitaxel-induced neuropathy. Paclitaxel (4.5 mg∙kg^−1^, Bristol-Myers Squibb, USA), vehicle (saline), or minoxidil (5, 25 or 50 mg∙kg^−1^, SC-200987A, Santa-Cruz, USA) was injected intraperitoneally (*i.p.*) on four alternative days (days 0, 2, 4, and 6). Von Frey filament (Part #2390, IITC Inc., CA) for mechanical hyperalgesia, tail immersion assay (water temperature: 48–49 °C) for thermal sensitivity and grip tests (BIO-EVF3, Bioseb Inc., USA) for grip strength were used as the methods described previously^11^. The baseline measurement of each behavior test was established prior to drug treatment, and five additional sessions were measured weekly.

### Mouse blood pressure, heart rate and locomotor measurement

Blood pressure (including systolic, diastolic and mean blood pressure) and heart rate of conscious mice were measured by using a non-invasive tail cuff plethysmography according to manufacturer’s instruction (Visitech Systems, Apex, NC, USA). Mouse locomotor activity by assessing the distance traveled in 5 min was automatically monitored by infrared light-beam animal activity monitors (ActiMot2, USA).

### Ultrastructure of the sciatic nerve

After neurobehavioral tests at the 5^th^ week following paclitaxel treatments, sciatic nerve samples were obtained from mice, fixed in 4% glutaraldehyde and post-fixed in 1% osmium tetroxide solution at 4 °C. These samples were then dehydrated in graded ethanol series and embedded in EMbed 812 (EMS-14120; Hatfield, PA, USA). The sciatic nerve sections (90 nm) were prepared, then observed and imaged with a transmission electron microscope (H7650, Hitachi, Japan).

### Trypan Blue Exclusion Assay

Trypan blue exclusion was used to determine the viability of cancer cells treated with minoxidil and paclitaxel for 48 hours. SiHa cells were seeded at a density of 1 × 105 cells/well onto 6-well plates overnight. And then, these cells were pre-treated with 1000 nM minoxidil for 24 h before paclitaxel treatment. After the treatments, the SiHa cells were stained with trypan blue solution (1 : 1) (Sigma-Aldrich, USA) and cells were counted in a Neubauer chamber with a light microscope. Cells viability was calculated using the following formula: viable cells (%) = (total viable cells/total cells (viable + dead)) × 100%.

### Tumor xenograft model

In mouse tumor xenograft models, the basolateral flank of 10-weeks-old NOD/SCID female mice (weight range: 20–25 g) were subcutaneously inoculated with 4 × 10^6^ human breast cancer MDA-MB-231 or 5 × 10^6^ cervical cancer SiHa cells. When tumor sizes had reached a size of approximately 150 mm^3^, mice were randomized to different subgroup to receive paclitaxel, minoxidil or vehicle on four alternative days. Tumor size was measured every six days for breast cancer and every four days for cervical tumor, respectively. Tumor volume (mm^3^) was calculated by using the formula: 0.5 × (the shortest tumor diameter)^2^ × (the longest tumor diameter).

### Hair growth experiment

The forty-two NOD/SCID female mice (10-weeks-old, weight range: 20–25 g) were randomly assigned to six groups, including control, paclitaxel, 25 mg∙kg^−1^ minoxidil, 50 mg∙kg^−1^ minoxidil, 25 mg∙kg^−1^ minoxidil/paclitaxel and 50 mg∙kg^−1^ minoxidil/paclitaxel group. Paclitaxel (4.5 mg∙kg^−1^), vehicle (saline), or minoxidil (25 or 50 mg∙kg^−1^) was injected *i.p.* on four alternative days (days 0, 2, 4, and 6) after used hair removal cream for epilation. The images of hair regrowth situations were recorded by digital camera (days 0, 13 and 19), and visual scoring of hair growth was performed on day 19 based on the scoring guideline shown in Fig. S4b. Hair length measurements were taken by plucking an average of 10 hairs on the regions following initial depilation after 19 days of treatment.

### Intracellular [Ca^2+^]_i_ measurement of DRG neurons

ND7/23 DRG cells (passage numbers: 10–15) were prepared and cultured for 3 days for the measurement of intracellular Ca^2+^ concentration ([Ca^2+^]_i_). ND7/23 DRG cells incubated with 2 μM Fluo-4/acetoxymethyl ester (Fluo-4/AM, F14201, Molecular Probes, USA) in DMEM medium at 37 °C for 30 min were washed three times with Ca^2+^ free HEPES solution (in mM: 145 NaCl, 5 KCl, 0 CaCl_2_, 10 glucose, 1 MgCl_2_, 5 HEPES, with an osmolarity of 297–300 mOsm and pH 7.40). The Fulo-4 was excited alternatively at 488 nm and the fluorescence intensity was analyzed at 520 nm. Then, [Ca^2+^]_i_ was calculated by an ImageXpressMicro automated wide-field fluorescent microscope (Molecular Devices, Sunnyvale, CA, USA). For [Ca^2+^]_i_ measurement, DRG neurons were stimulated with 2 μM thapsigargin (TG, Cayman Chemical, USA), recorded for 7 min subsequently.

### Immunohistochemistry

DRG tissues for assessing neuroprotective effect of minoxidil on paclitaxel-induced neuropathy were isolated at Day1, 4, or 7 after first paclitaxel injections. Sequentially, DRG tissues were fixed with 4% PFA for 4 hours and dehydrated with 30% sucrose in PBS overnight at 4 °C. For frozen sections, the samples were embedded in OCT (Leica, IL, USA) and sliced into 20 μm thick sections. These DRG tissue sections were washed with 0.05% Triton X-100 in PBS for 30 minutes and blocked with 3% BSA at room temperature for 1 hour. DRG tissue samples were co-stained with mouse anti-NeuN antibody (1:100; Millipore), mouse anti-NOS2 (1:100; Santa Cruz), mouse anti-Iba-1 (1:100; Santa Cruz), rabbit anti-Arginase-1 (1:100; Santa Cruz), rabbit anti-Iba-1 (1:100; Abcam) and rat anti-CD68 antibody (1:100; Novus, USA) at 4 °C overnight. Then, these samples were washed by PBS and incubated in secondary antibodies (Alexa-488 and Alexa-594 1:200; Invitrogen, CA) at room temperature for 1 hour. Immunofluorescence images of DRG tissues were acquired using a 40x objective by Olympus FV1000 confocal microscope (Olympus, Japan).

### Ethics statement

Animal care and experimental procedures were approved by the institutional animal ethics committee of National Cheng Kung University (approved IACUC number: 103243) and carried out accordance with Animal Care Guidelines.

### Statistical analysis

For all animal behavioral test at least 7 mice, tumor xenograft models 5 mice and hair growth experiment 7 mice were randomized to each group. Results are expressed as the mean ± SEM. Differences between groups were compared using a two-tailed Student’s t-test or a two-way ANOVA followed by the *Bonferroni post-hoc* analysis using statistics software SPSS Statistics 15.0 (IBM SPSS Statistics, USA). The criterion for statistical significance was P < 0.05. Experimenters were blinded to all drug treatments.

## Additional Information

**How to cite this article:** Chen, Y.-F. *et al*. Minoxidil is a potential neuroprotective drug for paclitaxel-induced peripheral neuropathy. *Sci. Rep.* **7**, 45366; doi: 10.1038/srep45366 (2017).

**Publisher's note:** Springer Nature remains neutral with regard to jurisdictional claims in published maps and institutional affiliations.

## Supplementary Material

Supplementary Figures
