# Supplementary material for: Minoxidil is a potential neuroprotective drug for paclitaxel-induced peripheral neuropathy
Source: Sci Rep. 2017 Mar 28;7:45366. doi: 10.1038/srep45366 (PMC5368986; doi:10.1038/srep45366)
Supplement: Supplementary Figures [file srep45366-s1.pdf]

Minoxidil is a potential neuroprotective drug for paclitaxel-induced peripheral neuropathy

Yi-Fan Chen, Li-Hsien Chen, Yu-Min Yeh, Pei-Ying Wu, Yih-Fung Chen,
Lian-Yun Chang, Jang-Yang Chang and Meng-Ru Shen

Supplementary Fig. S1: The effects of minoxidil on blood pressure, heart rate and locomotor function

Supplementary Fig. S2: The neuroprotective effects of minoxidil in a mouse model

Supplementary Fig. S3: The therapeutic potential of minoxidil in mouse model

Supplementary Fig. S4: The protective effect of minoxidil on paclitaxel-induced nerve degeneration

Supplementary Fig. S5: Minoxidil exhibits inhibitory effects on cell viability in SiHa cervical cancer cell line

Supplementary Fig. S6: Minoxidil enhances anti-tumor effect of paclitaxel in cervical cancer in vivo

Supplementary Fig. S7: Index of hair growth visual scoring guide in NOD-SCID mice

Supplementary Fig. S8: The time course of macrophage infiltration in mouse dorsal root ganglion (DRG) after administration of paclitaxel

Supplementary Fig. S9: Minoxidil inhibits the macrophage infiltration in paclitaxel-treated dorsal root ganglia (DRG)

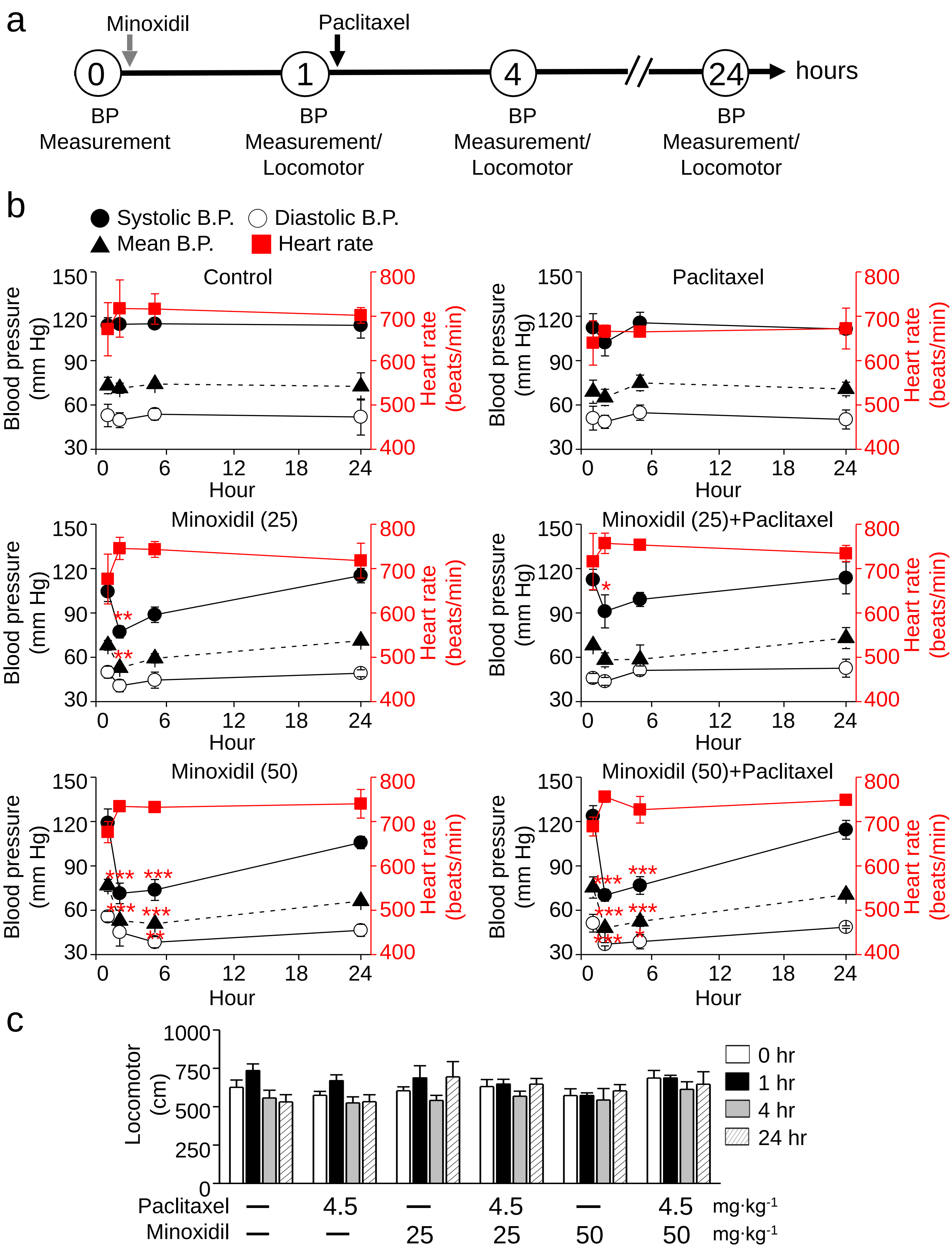

Figure S1. The effects of minoxidil on blood pressure, heart rate and locomotor function. (**a**) Protocols showing the measurement of blood pressure, pulse and locomotor activity in mouse model. (**b**) Time course on monitoring blood pressure, heart rate and (**c**) locomotor activity after minoxidil (25 or 50 mg·kg^{-1}) and paclitaxel (4.5 mg·kg^{-1}) treatment. Each value represents mean ± SEM from at least 4 different mice. * P < 0.05; **P < 0.01; ***P < 0.001 compared to baseline, by two-way ANOVA. B.P., blood pressure.

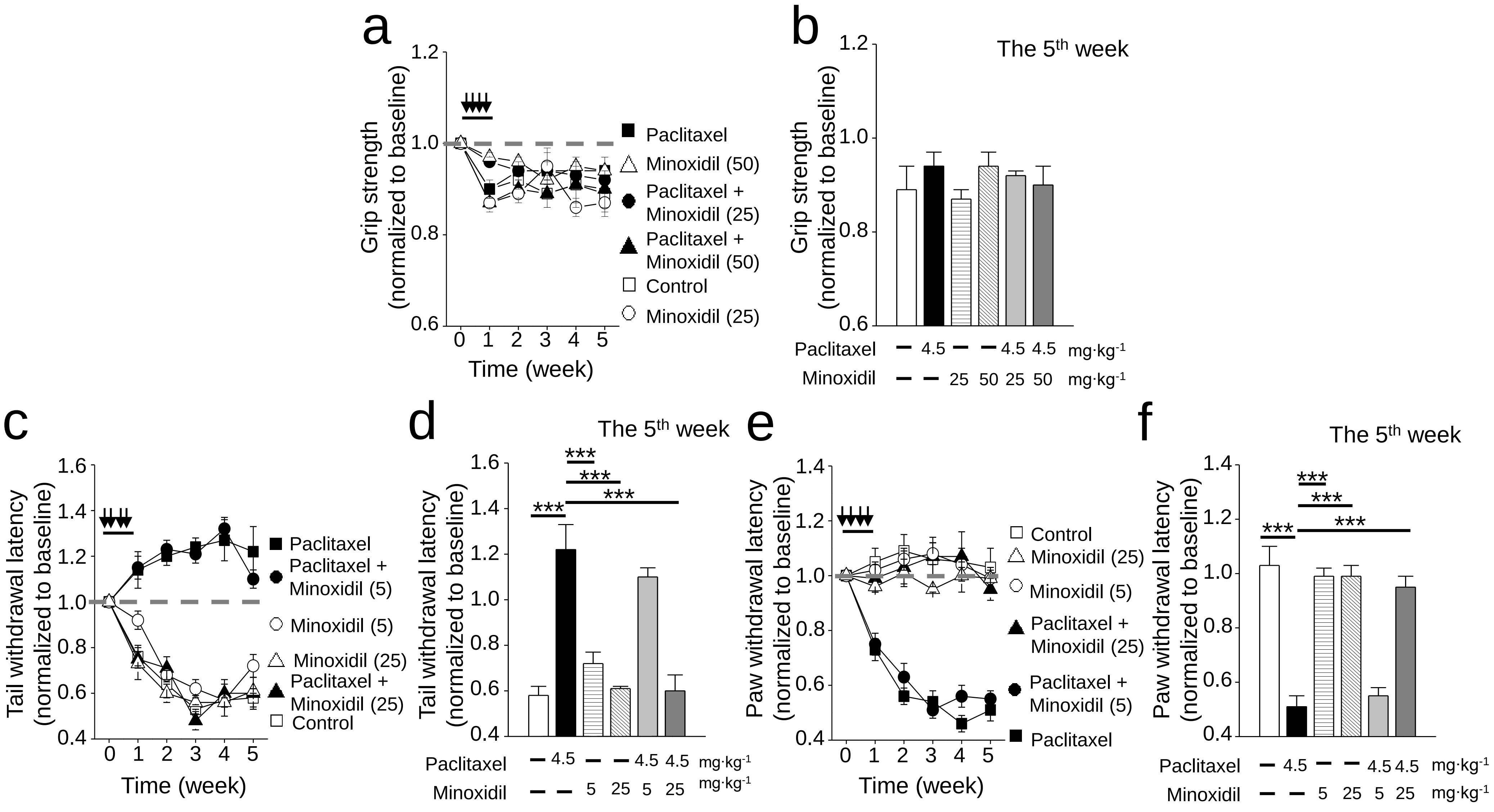

Figure S2. The neuroprotective effects of minoxidil in a mouse model. (**a**) Grip strength test to measure neuromuscular function. Y axis, normalized muscular strength from grasping wire to generating maximal muscle force. Black arrow, drug infusion. (**b**) Quantitative analyses of grip strength at the 5th week. (c-f), Lower dose of minoxidil (5 mg·kg^{-1}) showed no protective effect on paclitaxel-induced neuropathy. (**c**) Tail immersion test to assess thermal sensation. Y axis, normalized latency from tail immersion to tail withdrawal. Black arrow, drug infusion. (**d**) Quantitative analyses of tail immersion at the 5th week. Each value represents mean ± SEM (n = 7-9, per group). ***P < 0.001 versus paclitaxel treatment, by two-way ANOVA. (**e**) von Frey filament test to detect allodynia. Y axis, normalized pressure from touch to paw withdrawal. Black arrow, drug infusion. (**f**) Quantitative analyses of von Frey filament test at the 5th week. Each value represents mean ± SEM (n = 7-9, per group). ***P < 0.001 versus paclitaxel treatment, by two-way ANOVA.

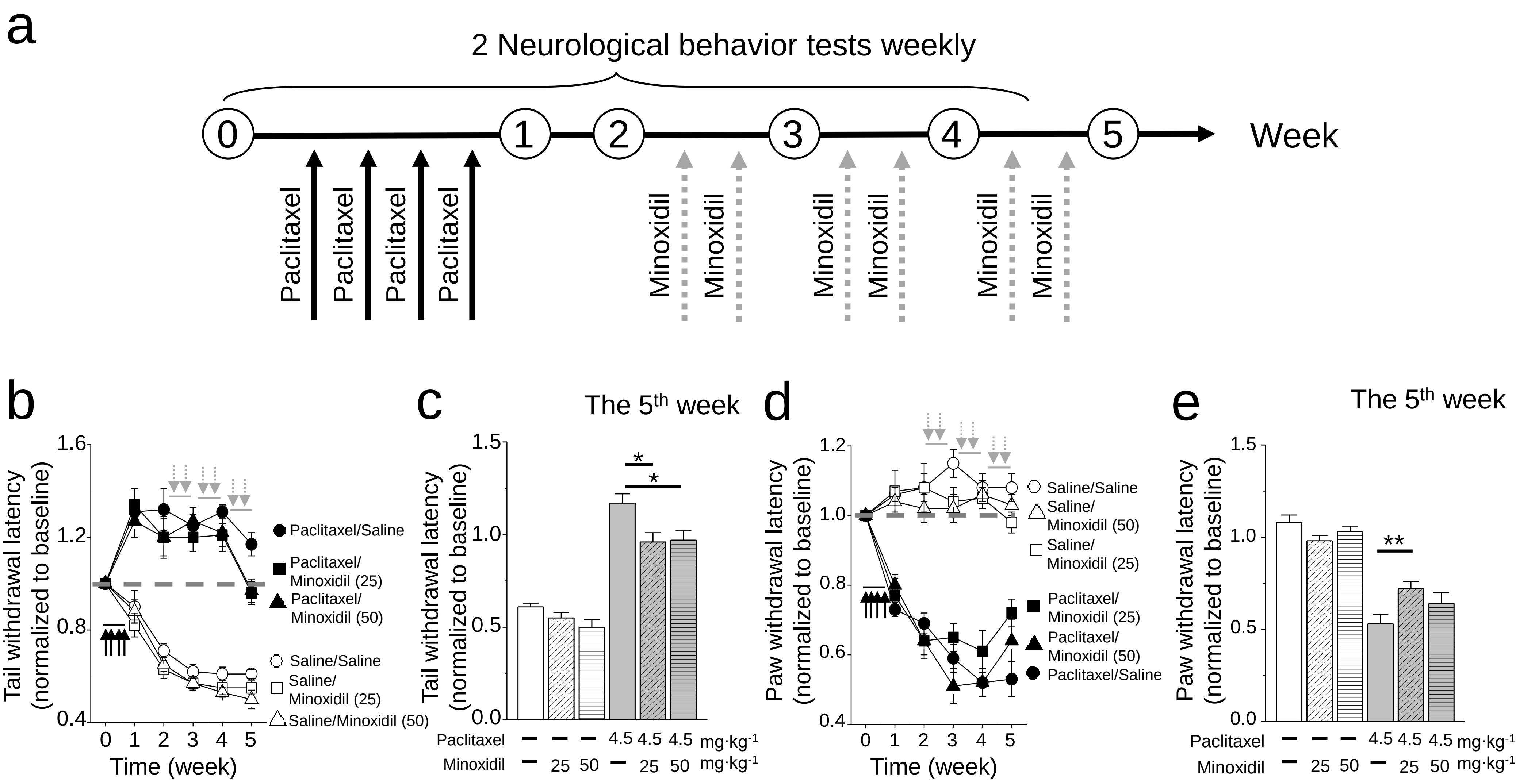

Figure S3. The therapeutic potential of minoxidil in mouse model. (**a**) Protocol showing drug administration and behavioral tests in mouse model. (b-e), minoxidil showed therapeutic potential on paclitaxel-induced neuropathy. (**b**) Tail immersion test to assess thermal sensation. Y axis, normalized latency from tail immersion to tail withdrawal. Black arrow, paclitaxel or saline infusion. Gray dashed arrow, minoxidil or saline infusion. (**c**) Quantitative analyses of tail immersion at the 5th week. Each value represents mean ± SEM (n = 7-9, per group). *P < 0.05 versus paclitaxel treatment, by two-way ANOVA. (**d**) von Frey filament test to detect allodynia. Y axis, normalized pressure from touch to paw withdrawal. Black arrow, paclitaxel or saline infusion. Gray dashed arrow, minoxidil or saline infusion. (**e**) Quantitative analyses of von Frey filament test at the 5th week. Each value represents mean ± SEM (n = 7-9, per group). **P < 0.01 versus paclitaxel treatment, by two-way ANOVA.

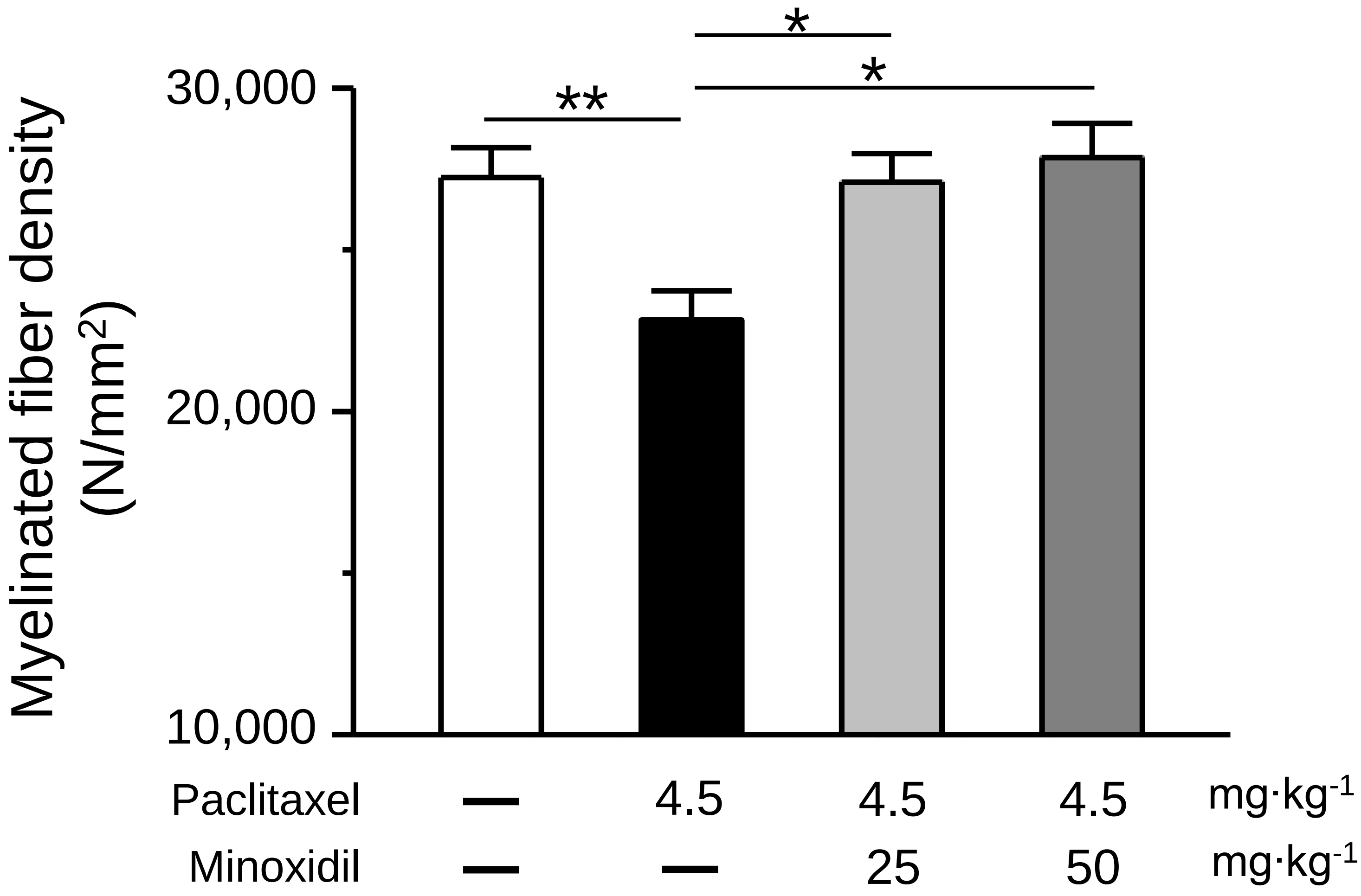

Figure S4. The protective effect of minoxidil on paclitaxel-induced nerve degeneration. Quantification of myelinated fiber density in the sciatic nerves revealed that paclitaxel-induced axon loss was improved by minoxidil. Data are shown as mean ± SEM for at least 4 different samples. * $P< 0.05$; ** $P< 0.01$ versus paclitaxel group, by two-way ANOVA.

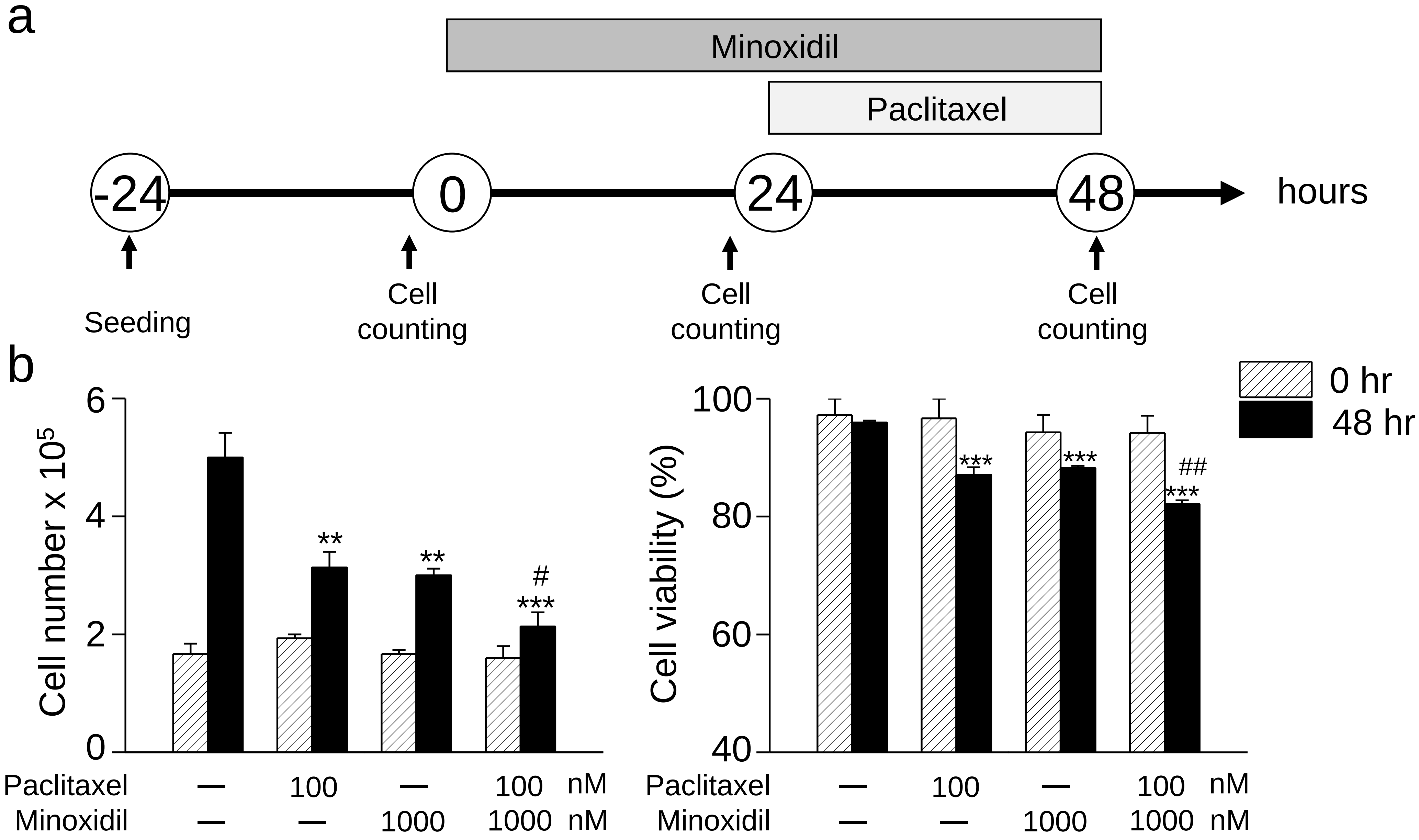

Figure S5. Minoxidil exhibits inhibitory effects on cell viability in SiHa cervical cancer cell line. (**a**) The protocol showing drug treatment and the measurement of cell proliferation. (**b**) Cell number (left) and viability (right) were analyzed after 48 hr incubation with 1000 nM minoxidil or 100 nM paclitaxel. Each value represents the mean ± S.E.M. from at least 3 different experiments. **P < 0.01; ***P < 0.001 compared to vehicle-treated cell; #P < 0.05; ##P < 0.01 compared to paclitaxel-treated group, by two-way ANOVA.

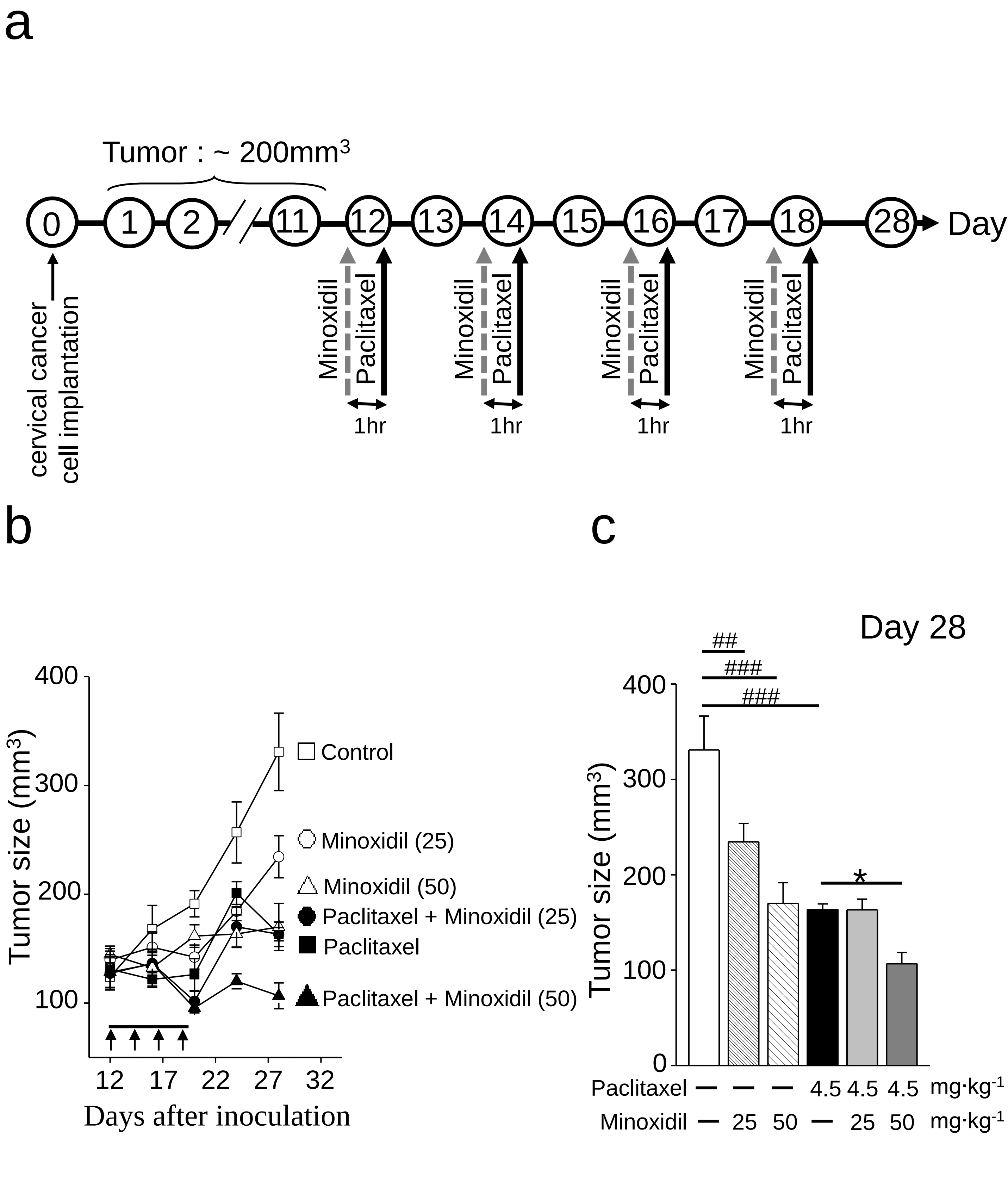

Figure S6. Minoxidil enhances anti-tumor effect of paclitaxel in cervical cancer *in vivo*. (**a**) Protocol showing drug administration in the tumor xenograft model. (**b**) Female NOD/SCID mice bearing tumor xenograft of SiHa cells were intraperitoneally injected every other day (arrows) with control saline, paclitaxel (PTX, 4.5 mg·kg^{-1}) or minoxidil (25 or 50 mg·kg^{-1}) prior to paclitaxel treatment from the 12th day post-inoculation (n = 5, per group). (**c**) Quantitative analyses of tumor volume at the day 28th post-inoculation. Columns, mean ± SEM (n = 5, per group); *P < 0.05 versus paclitaxel treatment; $^{##}$P < 0.01; $^{###}$P < 0.001 versus saline treatment, by two-way ANOVA.

a

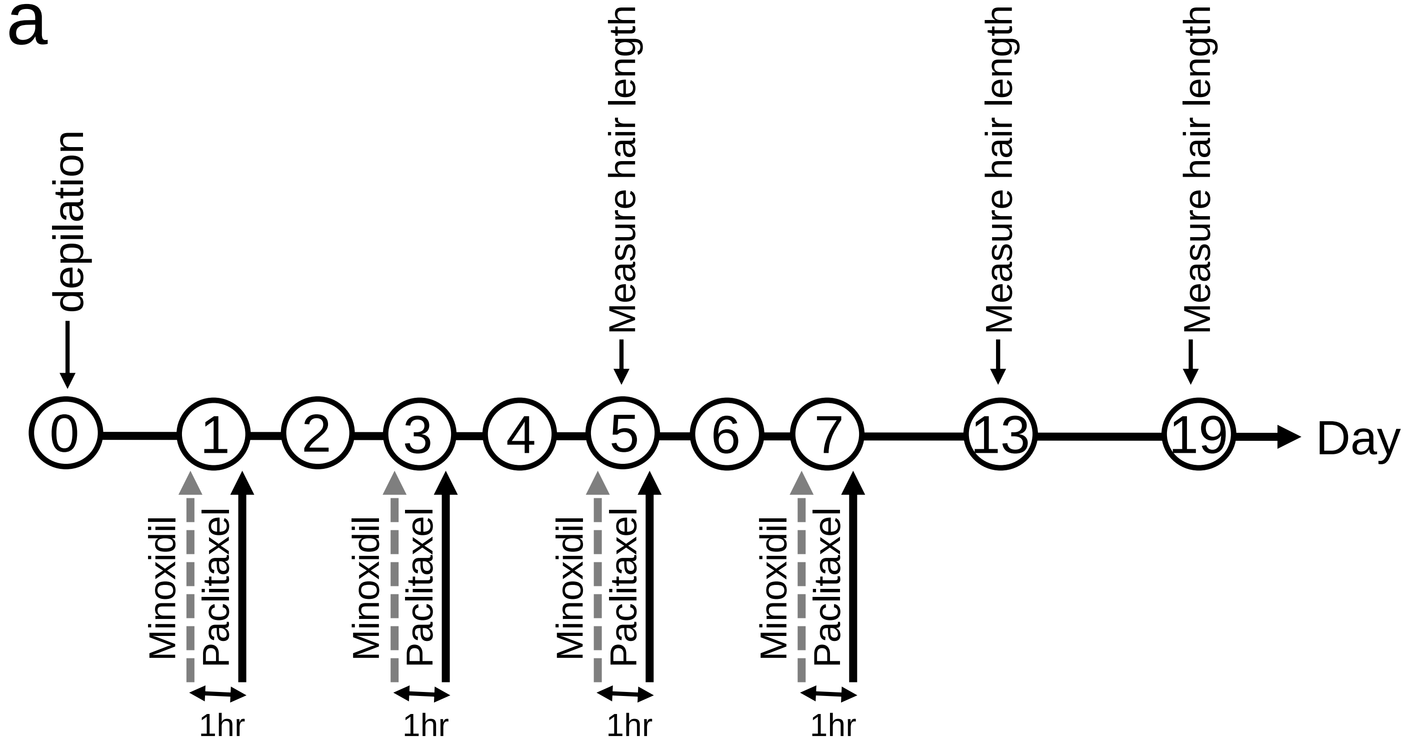

b

Grade	Condition	Pattern
1	No change	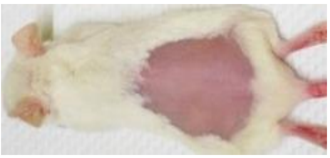
2	Skin become white	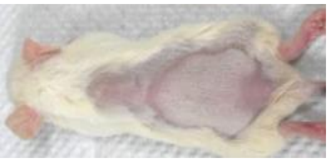
3	>30% of shaved area shows hair growth	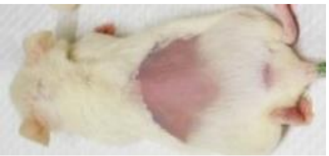
4	>60% of shaved area shows hair growth	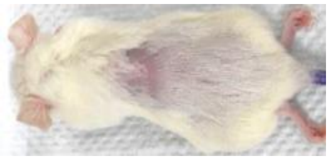
5	>90% of shaved area shows hair growth	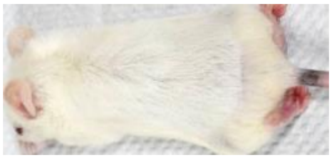

Figure S7. Index of hair growth visual scoring guide in NOD-SCID mice. (**a**) Time line showing the experimental design. The backs of mice were imaged and the regrowth of hair was quantified by measuring the length of plucked hairs at Days 0, 13 and 19 after epilation. (**b**) Hair growth quantification subdivided into five grades.

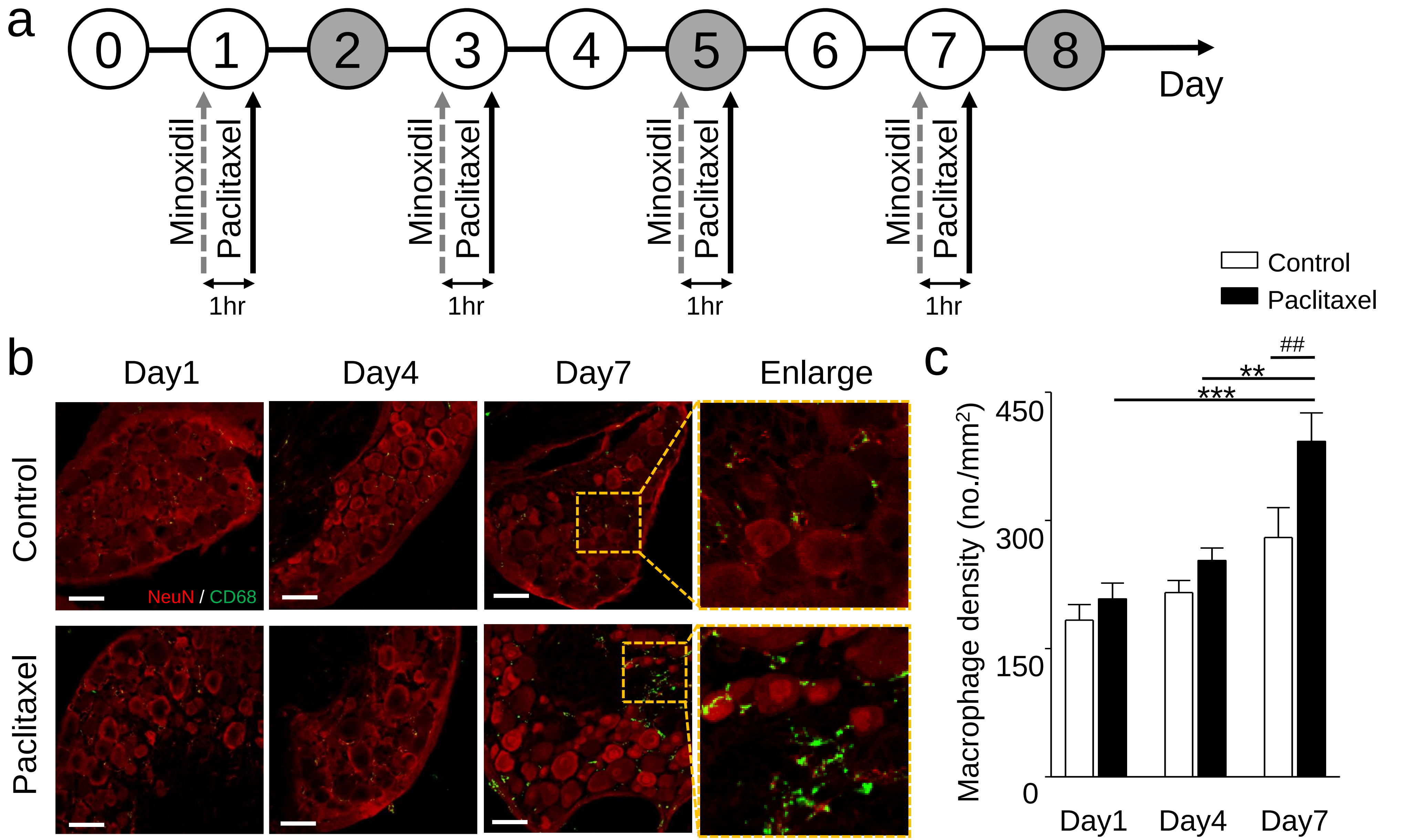

Figure S8. The time course of macrophage infiltration in mouse dorsal root ganglion (DRG) after administration of paclitaxel. (**a**) Protocol showing the experimental design. DRG neurons were isolated at Day 1, 4, or 7 after first paclitaxel or minoxidil injection for image studies. (**b**) DRG neurons are visualized by NeuN staining (red) and macrophages are labeled by CD68 (green). (**c**) Paclitaxel upregulates macrophage expression in the DRG with a time-dependent manner. Each value represents mean ± SEM in each group (n = 6). $^{\#\#}P < 0.01$ versus saline treatment; $^{**}P < 0.01$; $^{***}P < 0.001$ versus corresponding paclitaxel group on the 7th day, by two-way ANOVA. Scale bar, 50 μm.

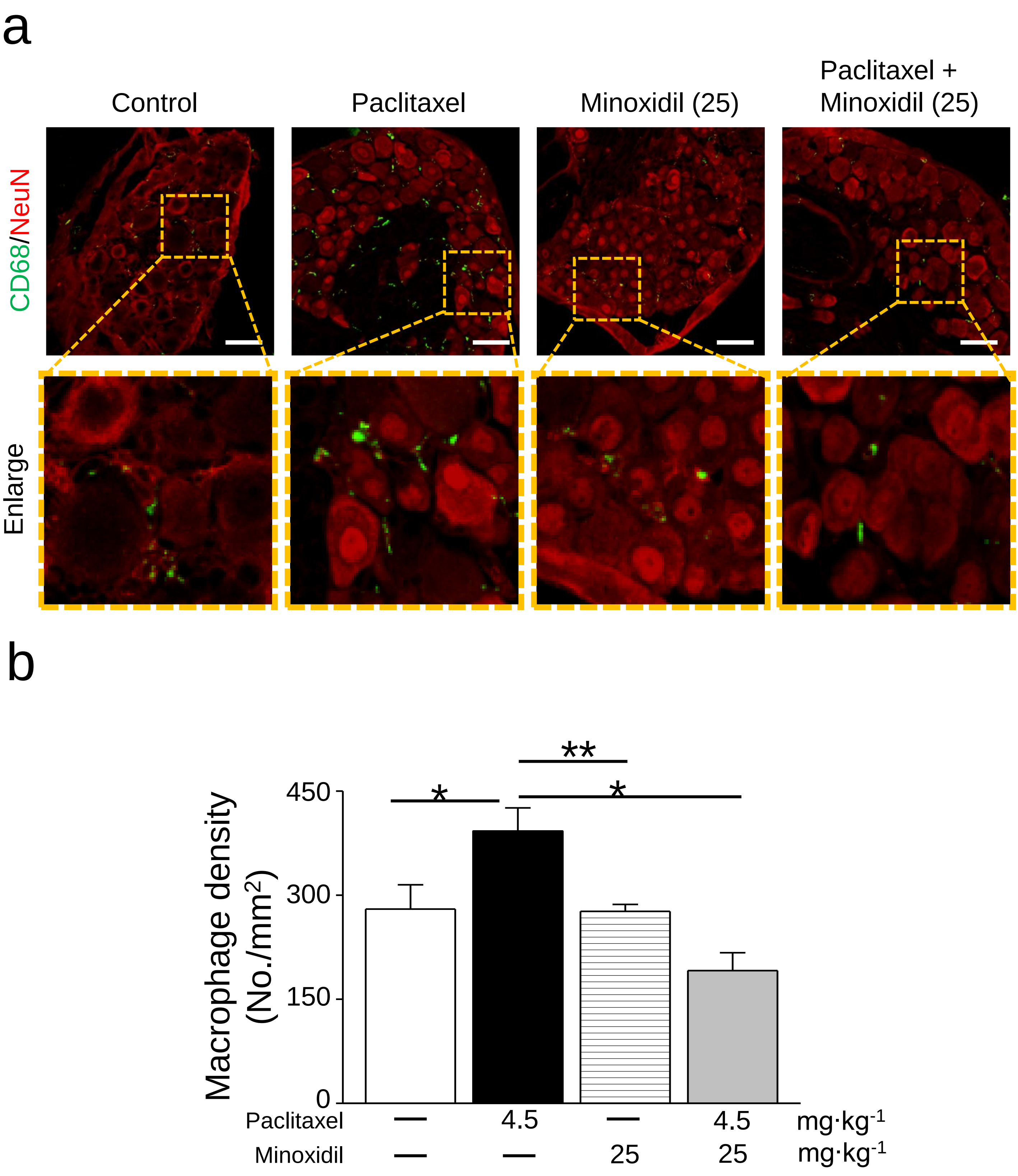

Figure S9. Minoxidil inhibits the macrophage infiltration in paclitaxel-treated dorsal root ganglia (DRG). (**a**) Double-staining of NeuN (neuron nuclei maker, red) and CD68 (macrophage maker, green) in DRG neurons on the 7th day after first paclitaxel (4.5 mg · kg^{-1}) or minoxidil (25 mg · kg^{-1}) prior to paclitaxel treatment. Scale bar, 50 μm. (**b**) Quantitative analyses of macrophage density in DRG neurons on the 7th day of paclitaxel treatment. Each value represents mean ± SEM of at least 6 different samples. *P < 0.05; **P < 0.01 versus paclitaxel treatment, by two-way ANOVA.
